# Updated Insights into Probiotic Nut-Based Dairy Alternatives: Microbiological, Antioxidant, and Sensory Aspects

**DOI:** 10.3390/foods15142505

**Published:** 2026-07-15

**Authors:** Ioanna Mantzourani, Vasiliki Adamopoulou, Argyro Bekatorou, Stavros Plessas

**Affiliations:** 1 Department of Chemistry, University of Patras, 26504 Patras, Greece; adamopoul_v@upatras.gr (V.A.); abekatorou@upatras.gr (A.B.); 2 Laboratory of Food Processing, Department of Agricultural Development, Democritus University of Thrace, 68200 Orestiada, Greece

**Keywords:** plant-based milk alternatives, nuts, probiotics, fermentation, antioxidant activity, lactic acid bacteria, nutritional value, sensory properties

## Abstract

Plant-based “milks” have gained increasing attention as functional beverages due to their nutritional properties, consumer acceptance, and potential for probiotic fermentation. Lactose intolerance, milk allergies, and growing interest in sustainable plant-based diets have contributed to the rapid expansion of nut-based beverage (NBBs) markets. This review summarizes recent advances in processing and nutritional characteristics of NBBs as dairy alternatives. Although rich in bioactive compounds, including antioxidants, minerals, unsaturated fatty acids, and vitamins, NBBs generally exhibit lower nutritional density than bovine milk, particularly in protein content and mineral bioaccessibility due to antinutritional compounds present in plant matrices. Fermentation with lactic acid bacteria (LAB) can confer probiotic properties, increase the bioaccessibility of bioactive compounds, and improve sensory properties by reducing off-flavors and promoting the formation of desirable aromas. Pistachio- and almond-based beverages generally exhibit more consistent fermentation performance, favoring probiotic growth and sensory improvement, whereas coconut- and walnut-based milks often require additional stabilization strategies due to limited emulsifying capacity or oxidative instability. Overall, fermented NBBs represent a promising category of functional foods with increasing technological and nutritional relevance. Future research should focus on optimizing starter cultures and processing conditions, improving scalability and industrial feasibility, and elucidating microbial/matrix interactions through integrated omics approaches.

## 1. Introduction

For many decades, cow’s milk and other milks of animal origin have been considered highly nutritious for human consumption. Nevertheless, contemporary concerns, primarily related to lactose intolerance, food allergies, vegetarianism, religious beliefs, and alternative lifestyles, have driven the demand for plant-based milk alternatives (PBMAs) [1,2].

In this context, PBMAs have been produced based on cereals, pseudo-cereals, legumes, seeds, and nuts [1,2]. Because of their origin, these products are more commonly classified as “dairy alternatives”, “drinks” or “beverages” rather than “milks” [3]. Terminology is also driven by regulatory frameworks, such as the European Regulation (EU) No 1308/2013, which reserves the term “milk” exclusively for mammary secretions [4]. While terms such as “plant-based milks” or “mylk” are commonly employed in marketing and everyday language to emphasize their role as substitutes for dairy milk, European legislation requires the use of non-dairy terminology for these products. In particular, in 2017, the restriction of dairy-related designations for non-animal-derived products was reinforced in the EU, permitting only a limited number of traditional exceptions in order to minimize potential consumer confusion [4,5]. Despite these regulatory limitations, the plant-based beverage (PBB) sector has experienced substantial commercial growth. Recent reports indicate that PBBs constitute one of the fastest-growing categories within the global food industry. This expansion has been largely attributed to increasing consumer interest in ethical consumption patterns, environmental sustainability, and health-conscious dietary choices, alongside ongoing improvements in product formulation and sensory quality. The global market, valued at approximately $2.8 billion in 2022 and projected to reach $7.3 billion by 2032, corresponds to a compound annual growth rate (CAGR) of 10.3% [6]. These trends highlight the progressive transition of PBBs from niche products to widely consumed dietary alternatives. PBBs are generally recognized as functional foods and nutraceuticals, largely due to their high content of minerals, vitamins, dietary fiber, and antioxidants [3,6]. While some (e.g., soybean, oat, rice, almond, coconut, and cashew) have been extensively studied for their technological properties, others (such as pistachio, walnut, lupin, hazelnut, macadamia, tiger nut, peanut, and chickpea) require further thorough investigation [1,3].

Furthermore, the processing methods employed for PBB production affect both the quality and quantity of several bioactive compounds of significant importance for consumers’ health [7]. At this point, it should be stated that nut-based beverages (NBBs) often exhibit lower nutritional value in comparison with their respective raw nuts, as a result of the production process [8]. Processes like soaking, roasting, milling, and de-oiling may alter the composition of the final product [2].

The present review critically examines the production processes and nutritional characteristics of NBBs, with particular emphasis on almond, coconut, hazelnut, pistachio, and walnut-derived products. In addition, recent advances in the functional enhancement of these beverages through probiotic incorporation are discussed, alongside their antioxidant properties and sensory characteristics.

## 2. Production Processes and Nutritional Properties of Nut Beverages

### 2.1. Manufacturing Stages: From Raw Material to Aqueous Extract

The industrial production of NBBs aims to convert solid plant matrices into stable colloidal dispersions with physicochemical and sensory properties comparable to those of bovine milk [9]. In general, the manufacturing process can be classified into two principal approaches: (i) conventional processing, which relies on mechanical disintegration, filtration, and thermal stabilization, and (ii) advanced processing, which integrates emerging technologies such as high-pressure homogenization, thermosonication, and enzymatic biotransformation in order to improve the physicochemical stability and preserve the nutritional quality. These processing stages collectively describe the transformation of raw almonds, coconuts, hazelnuts, pistachios, and walnuts into functional aqueous extracts suitable for beverage formulation [6,7,8].

#### 2.1.1. Pretreatments and Nut-Specific Processing

The initial preparation of the raw material is vital for determining the sensory and nutritional quality of the produced liquid extract. Raw materials may be supplied shelled or unshelled; in the latter case, shelling/dehulling is performed, often after soaking in hot water to facilitate shell removal [8]. For almonds and hazelnuts, thermal pretreatments such as roasting (typically at 95–100 °C) are frequently employed to develop desirable aromatic profiles by reducing concentrations of benzaldehyde and pyrazines [8,10]. Walnuts require specialized pellicle removal treatments using acidic (e.g., 2% citric acid) or alkaline (e.g., 1% NaOH) solutions to reduce bitterness caused by tannins and other phenolic compounds [8]. For coconuts, the process typically involves blanching or steam cooking for enzyme inactivation and microbial reduction prior to grinding [10].

#### 2.1.2. Aqueous Extraction

The conversion of nuts into a milky liquid is primarily achieved through two distinct approaches: the wet process and the dry process. The wet process involves soaking the raw materials to promote swelling and softening, followed by wet milling with the addition of water [3,8]. For coconut, water is typically added at 80 °C, whereas for pistachios, the extraction yield is optimized by adjusting the pH (in the range 6.5–8.5) and blending time (20–30 min) [10,11]. In contrast, the dry process involves dry grinding the material into a fine flour, which is then extracted in water. Extraction efficiency for all nuts can be enhanced by increasing temperature or pH and by employing enzymatic hydrolysis. Enzymes such as proteases, amylases, and cellulases may be used to hydrolyze proteins, starch, and cell wall polysaccharides, respectively, significantly improving extraction efficiency and the recovery of soluble solids and proteins [3].

Following these extraction approaches, nut-derived liquid products can be categorized based on their structural and compositional characteristics. “Nut milk” refers to an oil-in-water emulsion obtained through wet or dry processing of soaked or ground nut kernels with water, followed by separation of insoluble fractions [1,3,6,8,9]. “Nut beverage” represents a broader commercial and regulatory category encompassing all drinkable formulations made from nuts, including emulsified, diluted, and processed systems intended for consumption [3,4,7,11]. “Nut juice” is generally defined as the liquid fraction obtained by pressing raw nut tissue with minimal dilution, although this term is less consistently standardized across the literature [3,7]. “Nut water” refers to the naturally occurring liquid present in immature nuts, and is the least standardized term among nut-derived liquids [3,7].

#### 2.1.3. Formulation and Stabilization

Following extraction, the produced slurry is subjected to filtration, decantation, or centrifugation to remove coarse insoluble particles, such as fiber and cellular materials. The resulting filtered extract is then formulated with ingredients that may include sweeteners, flavorings, colorings, salt, oils, and stabilizers. To bridge the nutritional gap with cow’s milk, fortification with vitamins (A, B12, D2, E) and minerals, mainly calcium (Ca), is widely practiced [3,8,10].

Stability can be maintained through the incorporation of stabilizing agents, such as carrageenan, rice or tapioca starch, gellan gum, xanthan gum, guar gum, corn dextrin, etc. [3]. Hydrocolloids and emulsifiers are commonly used to improve the physical stability of NBBs through complementary mechanisms. Emulsifiers such as lecithin stabilize oil-in-water emulsions by reducing interfacial tension at the oil–water interface, whereas hydrocolloids primarily increase the viscosity of the continuous phase, thereby reducing particle sedimentation and gravitational phase separation. Depending on the beverage formulation, these additives may be used individually or in combination to improve viscosity, emulsion stability, and overall product stability [3,8,9,12].

#### 2.1.4. Homogenization and Preservation

Homogenization is a critical step for reducing particle size and preventing phase separation. Almond milk typically undergoes ultra-high-pressure homogenization (UHPH) at 350 MPa and 85 °C to increase stability [10]. The homogenization of hazelnut milk at 100 MPa provides better stability than 150 MPa, as higher pressures can decrease protein solubility and increase the solid particle sedimentation value [12].

Preservation is commonly achieved through thermal treatments such as pasteurization or ultra-high temperature (UHT) processing. However, current trends increasingly favor non-thermal or hybrid technologies to better preserve the product’s bioactive compounds. Emerging methods, including thermosonication for almond milk and UHPH for hazelnut milk, have been shown to better retain amino acids and maintain the physicochemical stability compared with conventional heat treatments [7,8,10,12].

#### 2.1.5. Packaging, Dehydration, and Storage

Regarding the packaging and storage of NBBs, the selection of an appropriate containment system is critical for maintaining product quality and extending shelf-life. NBBs are commonly packaged in multilayer carton systems or plastic bottles. In addition to their liquid form, these products can also be processed into stable powders via spray-drying or drum-drying, enabling reconstitution into the final beverage. However, liquid NBBs must be adequately stabilized prior to dehydration to ensure a consistent and high-quality reconstituted product. For extended shelf-life and optimal physicochemical stability, aseptic packaging combined with refrigerated storage at 4 °C is required to prevent microbial spoilage [3,8].

### 2.2. Impact of Processing on Bioactive Compounds

The transition from raw materials to an aqueous extract involves mechanical, thermal, and chemical stresses that significantly alter the bioactive profile of NBBs. While processing is essential for safety and stability, it often leads to a marked dilution of nutrients compared with whole nuts, as extraction and filtration steps inherently reduce the concentrations of proteins, fats, and micronutrients. For instance, the mineral content of NBBs is frequently lower than that of cow’s milk, a gap that may be further exacerbated during processing steps such as soaking, bleaching, or decantation, where water-soluble vitamins and essential minerals may be lost [2,3,7].

The retention of antioxidants is similarly sensitive to specific pretreatments. For example, in walnut processing, since the majority of antioxidants are concentrated in the skin, peeling can result in a loss of over 90% of the nut’s total antioxidant activity. Similarly, in the production of hazelnut milks, a 42% decrease in total phenolic content (TPC) has been reported, largely due to the loss of hydrophobic phenolics that are not efficiently partitioned into the aqueous phase [8]. In addition, thermal processing, although necessary for pathogen and enzyme inactivation, can cause a considerable reduction in nutritional value and degradation of heat-sensitive vitamins [3,13].

However, processing can also be transformative rather than destructive. For example, roasting has been shown to alter the sterol profile of almond beverages, with reported increases in *β*-sitosterol-*β*-D-glucoside and decreases in stigmasterol depending on processing conditions [8].

Beyond chemical changes, the physical stability of NBBs is managed through the addition of stabilizers such as hydrocolloids (e.g., xanthan and guar gum). While these additives enhance viscosity and prevent phase separation without compromising functional quality, their use may raise considerations regarding gastrointestinal tolerance in certain individuals consuming plant-based formulations [14].

To mitigate these nutritional losses, emerging non-thermal technologies such as pulsed electric fields (PEF) and UHPH are being explored for microbial reduction while helping to preserve heat-sensitive antioxidants and vitamins [6]. Furthermore, certain thermal treatments such as boiling have been reported to improve the bioavailability of minerals, including Ca, depending on the food matrix and processing conditions [8].

Due to the inherent compositional differences from cow’s milk and the impact of processing steps, NBBs are commonly fortified with Ca, phosphorus (P), and vitamins E and D to improve their nutritional profile and better align it with consumer expectations [11].

An overview of the influence of specific pretreatment and processing parameters on the nutritional and structural properties of various NBB types is provided in Table 1.

### 2.3. Nutritional Composition and Comparison with Cow Milk

Despite being promoted as healthy, bioactive-rich alternatives, NBBs and other PBMAs often exhibit lower overall nutritional density than cow milk [8,15]. The comparative nutritional profiles of cow milk and the studied NBBs are summarized in Table 2.

Unlike the relatively standardized composition of cow milk, PBMAs display considerable variability depending on the plant source, the degree of water dilution, formulation practices, and processing technologies employed [1,3,6]. Cow milk is widely regarded as a nutritionally complete food due to its balanced composition of high-quality proteins (caseins and whey proteins), highly bioavailable minerals such as Ca and P, and essential vitamins, including B12 and D, which contribute to bone health and physiological maintenance [3,13,15].

From a macronutrient perspective, one of the most significant differences concerns protein quantity and quality. Cow milk typically contains approximately 3.3–3.5 g protein/100 mL with high biological value, whereas many NBBs, such as almond and coconut beverages, often provide less than 1 g protein/100 mL [8,11,15]. Moreover, plant proteins may be limited in certain essential amino acids, particularly lysine, methionine, or tryptophan, depending on the botanical source. Therefore, fortification strategies or blending different plant sources are often required to improve protein quality and nutritional adequacy [6,11].

However, PBMAs may offer advantages regarding lipid composition. In contrast to cow milk, which contains cholesterol and relatively higher levels of saturated fatty acids, most NBBs are naturally cholesterol-free and contain greater proportions of unsaturated fatty acids, including monounsaturated and polyunsaturated fatty acids (PUFAs) such as oleic and linoleic acids, which are associated with potential cardiovascular benefits [8,15].

The glycemic and mineral profiles of cow milk and PBMAs also differ substantially. The primary carbohydrate in cow milk is lactose, a disaccharide that requires the enzyme lactase (*β*-galactosidase) for digestion, making its consumption problematic for lactose-intolerant individuals, who represent a considerable proportion of the global population. In contrast, NBBs are naturally lactose-free. However, many commercial PBMAs contain added sugars, such as sucrose or fructose, to enhance sensory acceptance, which may raise concerns regarding caloric intake and oral health [8,16].

Regarding mineral composition, cow milk naturally provides a balanced Ca:P ratio of approximately 1.3–1.4, which is considered favorable for bone development and mineral metabolism. In comparison, PBMAs frequently exhibit highly variable and sometimes unbalanced mineral ratios, particularly when not adequately fortified. For example, almond-based beverages may present substantially elevated Ca:P ratios, potentially compromising optimal mineral bioavailability and bone health if nutritional fortification is not carefully designed [2,16].

Despite these nutritional limitations, NBBs represent important sources of bioactive compounds that are absent or present in lower quantities in cow milk, including dietary fiber, phenolic compounds, and unsaturated fatty acids. Almond beverages, for instance, are recognized as sources of *α*-tocopherol (vitamin E) and may contain prebiotic compounds, such as inulin, that can beneficially modulate gut microbiota composition [11,15]. Pistachio-based beverages are particularly rich in carotenoids, including lutein, as well as antioxidant phenolic compounds such as catechins [7,11,15]. In addition, specific nut matrices may provide targeted functional benefits; walnut beverages are characterized by elevated levels of omega-3 fatty acids, particularly *α*-linolenic acid, whereas coconut-based beverages contain lauric acid, which has been associated with antimicrobial and immunomodulatory properties [10,11].

Overall, although PBMAs cannot generally be considered direct nutritional substitutes for cow’s milk without appropriate fortification, their low lactose content, favorable fatty acid profile and antioxidant potential make them attractive functional food matrices. Their nutritional and functional value may be further enhanced through fermentation, which can improve nutrient bioavailability and promote the formation of beneficial bioactive compounds [3,8,11,12,13,14].

**Table 2:** Nutritional composition of nut beverages (per 100 mL).

Beverage Type	Energy (kcal)	Protein (g)	Fat (g)	Carbohydrates (g)	Fiber (g)	Key Bioactives Reported	Ref.
Cow milk						Caseins, whey proteins, bioactive peptides derived from digestion/processing (casokinins, lactoferricin)	[11,13,15]
All types	69.0–118.0	2.90–6.00	3.60–6.40	3.20–5.40			
Standard	60–70	3.2–3.5	3.2–4.0	4.7–5.0			
Almond	15–38	0.42–0.59	1.04–1.10	0.58–6.59	0.0–0.40	α-Tocopherol, arabinose, flavonoids, phytosterols	[3,11]
Coconut	50–92	0.59–2.0	4.12–6.0	3.75–9.41		Lauric acid, medium-chain triglycerides, α-linolenic acid, α-tocopherol	[11,13]
Hazelnut	70–74	1.0	7.3	1.0–3.0		β-Sitosterol, α-tocopherol, catechin, mono- and oligomeric flavan-3-ols	[7,11]
Pistachio	50–99	1.6–2.5	4.6–8.3	0.2–1.9	2.1–2.9	Tocopherols, β-sitosterol, phenolic compounds	[7,11]
Walnut	72–92	0.8–2.9	3.2–7.5	0.4–4.8		PUFAs, ellagitannins, phenolic antioxidants	[7,11]

Highly concentrated, minimally diluted, or specially fortified/high-solids products may contain much higher levels of fiber, protein and other nutrients. PUFAs: Polyunsaturated fatty acids.

### 2.4. Nut Beverages as Substrates for Probiotic Growth

The transformation of NBBs from simple dairy alternatives into functional foods is largely driven by their suitability as substrates for probiotic microorganisms. Although these beverages differ nutritionally from cow milk, they provide a matrix composed of lipids, proteins, and varying levels of carbohydrates that can be utilized during fermentation. Microbial fermentation is not only a preservation method but also a biotransformation process that can improve sensory quality, texture, shelf-life, and nutritional functionality through the production of organic acids and other bioactive metabolites [7,13].

Nut-based matrices, including almond, coconut, walnut, hazelnut, and pistachio beverages, have been widely investigated for their compatibility with lactic acid bacteria (LAB). Species such as *Lactiplantibacillus plantarum*, *Lacticaseibacillus rhamnosus*, *Lacticaseibacillus reuteri*, and *Streptococcus thermophilus* are commonly used in fermented PBBs, including NBBs, and are generally capable of maintaining viable cell counts (VCCs) at levels considered potentially probiotic [6–8 log colony-forming units (CFU)/mL], depending on formulation, processing conditions, and storage environment. However, survival is strongly matrix-dependent and often requires nutritional supplementation [7,11].

The addition of fermentable substrates such as simple sugars or prebiotic compounds (e.g., inulin) has been reported to improve probiotic growth and viability in plant-based systems by providing additional energy sources and enhancing stress tolerance [11]. Overall, successful fermentation of NBBs is closely linked to formulation design, including carbohydrate availability, protein content, and total solids, which influence microbial performance and stability [7,11].

Specifically, fermentation of coconut-based beverages has been shown to support the production of exopolysaccharides (EPS) by certain LAB, which may contribute to improved texture and potential functional properties such as antioxidant activity. Similarly, fermentation of walnut- and pistachio-based beverages has been associated with changes in volatile compound profiles and improved sensory characteristics, including reduction in raw nut off-flavors and development of more desirable aroma notes [7]. These effects are primarily attributed to microbial metabolism of lipids and amino acids during fermentation, although outcomes vary depending on strain and processing conditions.

Beyond sensory and technological improvements, probiotic fermentation may also enhance mineral bioaccessibility in nut-based matrices. LAB can produce enzymes such as phytases, which contribute to the degradation of phytic acid, a known antinutrient that reduces mineral absorption. This process, together with acidification and the formation of soluble mineral complexes, can improve the bioaccessibility of minerals such as iron (Fe), zinc (Zn), and Ca [8,12].

From a broader perspective, PBMAs are increasingly recognized as suitable carriers for functional ingredients and probiotics, although their nutritional composition is highly variable and often requires fortification to match dairy milk [9,10,12]. Structural design and formulation strategies are therefore critical for optimizing both microbial viability and nutritional performance in next-generation PBBs [9].

## 3. Probiotication of Nut Beverages, Antioxidant Activity and Sensory Properties

Fermented NBBs and other PBMAs represent the convergence of traditional fermentation practices and modern functional food development. Fermentation of plant matrices, including nuts and pseudocereals, has been widely explored as a strategy to improve technological functionality, sensory quality, and potential nutritional value. In particular, these substrates provide fermentable carbohydrates and bioactive precursors that can be metabolically modified by LAB and other starter cultures, supporting the development of novel plant-based dairy alternatives [14,15].

During fermentation, microbial enzymatic activity leads to the breakdown of complex macromolecules into smaller compounds, including organic acids, peptides, and other metabolites. These transformations may improve digestibility and contribute to changes in the nutritional profile of PBBs. In addition, fermentation can influence the presence, structural stability, and availability of bioactive compounds, particularly phenolic derivatives, vitamins, and peptides, which have been associated in the literature with antioxidant-related activity, although the magnitude of these effects is dependent on substrate composition, microbial strain, and processing conditions [17].

From a sensory perspective, fermentation is widely recognized as a key tool for improving the acceptability of PBBs, and plant-based substrates in general, through multiple complementary metabolic pathways. Carbohydrate fermentation generates organic acids and aroma-active metabolites, including diacetyl, acetoin, acetaldehyde, and acetic acid, which impart desirable buttery, creamy, and fermented notes. Simultaneously, lipid metabolism reduces off-flavor aldehydes (e.g., hexanal, nonanal, and octanal), responsible for green or beany odors, either by decreasing their precursors or converting them into less odor-active compounds. In parallel, microbial proteolysis releases peptides and free amino acids that serve as precursors for alcohols, aldehydes, esters, acids, sulfur compounds, and branched-chain aldehydes, thereby enhancing flavor complexity while reducing bitterness. These sensory improvements are highly strain- and substrate-dependent, highlighting the importance of selecting appropriate microbial cultures to optimize the aroma, taste, and overall acceptability of fermented PBBs [17,18].

Overall, comparative analyses between dairy milk and PBMAs highlight that while PBBs offer environmental and dietary diversification benefits, they often require technological optimization, such as fermentation and fortification, to improve their sensory and nutritional quality [15,16].

### 3.1. Almond Milk (Prunus dulcis)

Al Zahrani & Shori [19] evaluated the performance of four probiotic strains, namely *L. rhamnosus*, *Lactobacillus acidophilus*, *L. plantarum*, and *Lacticaseibacillus casei*, in almond milk, soy milk, and mixed almond/soy formulations during three weeks of refrigerated storage. All fermented beverages maintained VCCs at levels of approximately 6 log CFU/mL or higher throughout storage. In addition, increases in TPC and total flavonoid content (TFC) were observed after fermentation and during storage. Among the tested formulations, almond milk fermented with *L. rhamnosus* exhibited the highest TFC values [19].

Building upon these findings, a subsequent study by the same research group [20] investigated probiotic fermentation in hybrid almond/cow milk systems at different ratios (75:25, 50:50, and 25:75). The authors reported that these blended matrices were capable of sustaining probiotic populations, with VCCs ranging from approximately 5.9 to 6.8 log CFU/mL during refrigerated storage. Fermentation by *Lactobacillus* spp. was also associated with increased antioxidant activity across formulations. The study suggested that combining dairy and plant-based substrates may improve physicochemical stability and support probiotic viability in fermented beverages [20].

The development of synbiotic almond beverages has also attracted considerable interest. Muncey & Hekmat [21] investigated the incorporation of short-chain and long-chain inulin (2% and 5%, *w*/*v*) into almond beverages fermented with *L. rhamnosus* GR-1. All formulations maintained probiotic viability above 7 log CFU/mL during 30 days of refrigerated storage. The addition of short-chain inulin promoted greater microbial growth and faster acidification compared with long-chain inulin and control samples. Importantly, inulin supplementation did not adversely affect probiotic stability, supporting the feasibility of synbiotic almond-based beverages as non-dairy functional products [21]. This optimization of flavor profiles directly addresses the sensory hurdle in market adoption; by bridging the gap between raw plant notes and familiar fruit-yogurt profiles, fruit juice supplementation significantly increases consumer repeat-purchase intent, stabilizing the product’s market lifecycle [22,23].

Technological approaches aimed at improving probiotic stability have also been explored in almond-based systems. Lipan et al. [24] evaluated spray-dried almond milk fermented with *L. plantarum* ATCC 8014 and monitored probiotic survival during storage at 4 °C and 22 °C for eight months. Refrigerated storage effectively preserved probiotic viability, maintaining counts around 7 log CFU/g throughout the storage period. In contrast, samples stored at ambient temperature exhibited a progressive decline in viability, falling below recommended probiotic levels after six months. These findings highlight the importance of storage temperature for maintaining the functional quality of dehydrated probiotic almond products [24].

The incorporation of fruit-derived ingredients has also been investigated as a strategy to improve the nutritional and sensory properties of fermented almond beverages. Duran and Cevik [25] examined the effect of orange juice addition (10%, 20%, and 30%) on probiotic fermented almond milk inoculated with *L. acidophilus.* All formulations maintained high microbial populations, ranging from approximately 7.58 to 7.95 log CFU/mL. Furthermore, beverages containing 20% and 30% orange juice received higher sensory scores for overall acceptability compared with control formulations [25].

Additional studies have evaluated the use of *Bifidobacterium* spp. in fermented PBBs. Al Zahrani et al. [26] investigated the application of *Bifidobacterium longum* and *Bifidobacterium animalis* subsp. lactis in soy-based fermented beverages and reported viable probiotic populations ranging from approximately 6.5 to 7.0 log CFU/mL during storage. Fermentation was also associated with increased antioxidant activity in the treated samples [26].

Alternative starter cultures, including kefir consortia, have also demonstrated promising functionality in almond-based beverages. Lahrairi et al. [27] characterized kefir-fermented almond milk prepared from Moroccan *Prunus dulcis* varieties. Fermentation increased TPC values by approximately two- to three-fold and enhanced DPPH (2,2-diphenyl-1-picrylhydrazyl) radical scavenging activity to levels of approximately 62–66%. LAB populations reached approximately 8 log CFU/mL, while sensory evaluation indicated favorable consumer acceptance of the fermented beverages [27].

Finally, Kılınç et al. [28] comparatively evaluated almond-, soy-, and oat-based fermented beverages produced using *Streptococcus thermophilus*, *Lactobacillus delbrueckii* subsp. *bulgaricus*, *L. acidophilus* NCFM, and *Bifidobacterium lactis* HN019™. Most strains maintained viable populations above 6 log CFU/mL during 21 days of storage at 4 °C, although lower survival was observed for *B. lactis* in some formulations. Fermentation resulted in modest increases in antioxidant activity (0.72–0.75 mmol Trolox equivalents/L), while sensory analysis demonstrated acceptable consumer perception regarding flavor, odor, texture, acidity, and overall acceptability. Among the tested matrices, fermented almond- and oat-based beverages achieved the highest sensory scores during storage [28].

Table 3 summarizes the reported probiotic viability, antioxidant activity, and phenolic content of the aforementioned studies.

### 3.2. Coconut Milk (Cocos nucifera)

In addition to PBMAs, the utilization of coconut water as a fermentation substrate has demonstrated significant potential for the delivery of *Bifidobacterium* strains. Santos et al. [29] evaluated the performance of four specific isolates, *B. animalis* B-41406, *Bifidobacterium bifidum* B-41410, *Bifidobacterium breve* B-41408, and *Bifidobacterium longum* subsp. *infantis* B-41661, during a 24 h fermentation period. The resulting beverages maintained high microbial stability under refrigerated storage (4 °C), with VCCs above 6 log CFU/mL for up to 42 days. Beyond viability, the study incorporated a sensory evaluation to determine consumer preference among the different formulations. The results indicated that the beverage fermented with *B. bifidum* achieved the highest overall acceptance, highlighting the influence of strain selection on the sensory characteristics of fermented coconut water.

Han et al. [30] investigated the growth characteristics and antibacterial properties of eight probiotic strains in fermented coconut milk against *Streptococcus pyogenes*. Among them, *Streptococcus salivarius* ATCC 13419 showed the highest survivability rates. In particular, strains ATCC 13419 and K12 exhibited enhanced antibacterial activity against *S. pyogenes*, with inhibition rates of 60% and 67%, respectively. This antimicrobial efficacy is driven by the microbial bioconversion of coconut’s medium-chain fatty acids, such as lauric acid, into free fatty acids and monoglycerides, which destabilize pathogenic cell membranes [31].

Vitheejongjaroen et al. [32] examined the efficacy of *Lacticaseibacillus paracasei* MSMC 36-9 in fermenting coconut milk to produce a yogurt-type beverage. The viability of the strain in the fermented samples remained high, ranging between 12 and 13 log CFU/g throughout the 21-day storage period. Moreover, the antioxidant activity of the samples fermented with *L. paracasei* was higher than that observed in samples fermented with a commercial yogurt starter culture.

The development of functional coconut-based beverages has also been associated with the production of bioactive metabolites during fermentation. Goveas et al. [33] investigated the growth of *L. plantarum* SVP2 and its EPS production in coconut water supplemented with soybean and green gram extracts. VCCs remained stable during refrigerated storage for 7 days, at approximately 8 log CFU/mL, while EPS content decreased only slightly, from 64.17 g/L to 62.62 g/L. Beyond contributing to beverage structure and stability, the EPS fractions, in synergy with probiotic *L. plantarum*, enhanced the functional value of the product through documented antibacterial and anti-biofilm properties. High acceptance of flavor and texture was recorded; however, overall consumer acceptability was moderate, mainly because of the beverage color and slightly sour fermented odor.

Other strains examined for their viability in coconut juice included *L. casei* 393, *L. plantarum* ATCC 20174, *L. rhamnosus* ATCC 7469, and *Lactococcus lactis* IO-1, each used as a single starter culture [34]. An increase in phenolic compounds, antioxidant activity, and tannin content was observed during refrigerated storage. All probiotic strains remained viable throughout storage, with *L. lactis* IO-1 achieving the highest VCCs (8.4 log CFU/mL). No significant differences among samples were observed regarding taste, aroma, color, or appearance.

Furthermore, Aher et al. [35] developed a probiotic beverage by fermenting hibiscus tea with coconut water using *L. acidophilus* 10307. The VCCs reached 6.32 log CFU/mL after 28 days of storage at 4 °C. Phytochemical analysis revealed increased TPC (7.8–14 mg GAE/mL) and TFC (26.5–87.7 mg QE/mL). Sensory evaluation yielded a score of 6.9 on a 9-point hedonic scale. Additionally, the beverage exhibited strong antioxidant activity, effectively scavenging DPPH and H_2_O_2_ radicals.

Senjaliya and Georrge [36] applied two strains, *L. plantarum* CMGC2 and CMJC7, isolated from cow’s milk, for the fermentation of mixed coconut and carrot juice beverages. Both strains demonstrated adequate growth, exceeding 8 log CFU/mL after 48 h of incubation.

According to Kantachote et al. [37], mature coconut water serves as an ideal substrate for *L. plantarum* DW12, achieving VCCs of 8.4 log CFU/mL after 48 h of fermentation. This process significantly enhanced the beverage’s bioactive profile, yielding a TPC of 134 μg/mL and strong antioxidant activity, with ABTS [2′-azino-bis(3-ethylbenzothiazoline-6-sulfonic acid)] and DPPH radical scavenging activities of 75% and 55%, respectively. Although fermentation introduced a characteristic sour taste and aroma, the addition of 20% honey effectively balanced the flavor profile and substantially improved sensory acceptability, highlighting the beverage’s potential as a novel functional product.

Finally, Zhao et al. [38] used *L. plantarum* MWLp-4 for the production of high-viscosity fermented coconut milk. When combined with commercial starter cultures, this strain improved viscosity and sensory characteristics, demonstrating strong potential as a natural alternative to chemical thickeners and stabilizers commonly used in the food industry.

Table 4 summarizes the aforementioned studies in terms of strain viability, antioxidant activity, and phenolic content.

### 3.3. Hazelnut Milk (Corylus avellana)

Hazelnut milk is characterized by a nutrient-dense composition, including a high proportion of monounsaturated fatty acids, significant levels of vitamin E (α-tocopherol), and appreciable amounts of phenolic compounds [7,11]. These compositional attributes, together with its naturally creamy sensory profile, contribute to its suitability as a dairy alternative. From a technological perspective, hazelnut milk has been shown to support LAB fermentation and maintain probiotic viability, indicating its potential as a functional substrate in PBB systems [39]. However, because hazelnut milk has a lower baseline protein content and lower natural buffering capacity compared to almond or pistachio milks, acidification during fermentation tends to occur more abruptly, which can accelerate the decline of certain acid-sensitive probiotic strains during extended storage [40].

In this context, Kalkan & Balpetek Külcü [41] investigated the application of viili culture (a traditional Scandinavian ropy fermented milk culture) in hazelnut milk for the development of a functional PBB. The study reported successful LAB growth during storage, with VCCs reaching up to 7.91 log CFU/mL. However, sensory evaluation indicated that fermented hazelnut milk samples were less preferred compared to cow milk controls, highlighting formulation-dependent consumer acceptance.

Similarly, Bernat et al. [42] demonstrated that fermentation of hazelnut milk with *L. rhamnosus* GG resulted in VCCs of 7.9 log CFU/mL immediately after fermentation and 8.3 log CFU/mL after 28 days of refrigerated storage, indicating strong probiotic stability.

Gocer & Koptagel [43] evaluated kefir production using nut-based milks without additives or added sugars. During 30 days of storage at 4 °C, a decrease in total aerobic mesophilic, lactic acid, and acetic acid bacteria was observed, whereas yeast populations increased, reflecting a shift in microbial ecology typical of kefir fermentation systems. Among nut-based formulations, the lowest microbial reduction was observed in almond kefir, followed by walnut, peanut, cashew, and hazelnut kefirs. In a related study, Gocer & Koptagel [44] produced kefir beverages from mixed nut milks (cashew, hazelnut, walnut, and almond) and compared them with cow milk kefir. The results showed that hazelnut-based kefir provided an energy content of 73.71 kcal/100 g, while hazelnut kefir reached 74.89 kcal/100 g. Additionally, nut-based kefirs exhibited higher unsaturated fatty acid content and lower saturated fatty acid levels compared to cow’s milk kefir, indicating improved lipid nutritional quality.

Erem & Akyilmaz [45] developed a fermented creamy structure from whole hazelnut kernels using yogurt starter cultures combined with sucrose. The fermentation process improved textural properties, including gel strength, yield stress, and spreadability. LAB VCCs exceeded 6 log CFU/g, with only a slight decline observed during refrigerated storage at 4 °C.

Furthermore, Akalin et al. [46] produced probiotic frozen desserts based on plant-derived milks (almond, hazelnut, and lupine) using *Lacticaseibacillus acidophilus*. Throughout 90 days of storage, hazelnut-based formulations maintained VCCs between 3.22 and 6.19 log CFU/mL. TPC ranged from 42.30 to 44.65 mg GAE/g, while antioxidant activity varied between 43.77 and 72.17 mM. Among the formulations, almond/hazelnut blends demonstrated the highest probiotic viability, whereas lupine-containing samples exhibited superior antioxidant performance.

Maleki et al. [47] demonstrated that fermentation of hazelnut milk using kefir grains significantly enhances its functional properties. Under optimized conditions (26 °C and 8% inoculum), antioxidant activity increased markedly, with DPPH radical scavenging activity rising from 50.47% to 81.65%. Although TPC decreased during fermentation (91.75 mg GAE/100 mL after 48 h), overall bioactivity, texture, and sensory acceptability improved. Lower fermentation temperatures and inoculum levels were associated with better preservation of hazelnut aroma and reduced formation of undesirable sensory attributes.

Overall, these studies indicate that hazelnut milk represents a promising substrate for fermentation-driven development of functional PBBs with enhanced microbial stability, improved nutritional profiles, and variable but generally acceptable sensory characteristics.

Table 5 summarizes the included studies in terms of probiotic strain viability, antioxidant activity, and phenolic content.

### 3.4. Pistachio Milk (Pistachio vera)

Pistachio milk is characterized by a distinctive green coloration associated with its chlorophyll and carotenoid content, including lutein, as well as notable levels of antioxidant compounds. It contains appreciable amounts of high-quality proteins enriched in essential amino acids, along with phenolic constituents such as catechins and gallic acid [11,48]. These compositional features of pistachio milk, particularly its relatively high protein, amino acid (e.g., arginine and glutamic acid), and mineral contents, provide a suitable matrix for LAB fermentation and have stimulated increasing interest in its application for fermented functional PPBs [17,18,49,50].

During fermentation, pistachio-based beverages undergo notable physicochemical and sensory transformations driven by LAB metabolism. LAB activity contributes to the modification of volatile compound profiles, reducing undesirable raw nut-like aromas while promoting the formation of fermentation-associated flavor compounds, including acetoin and 2,3-butanedione, which are characteristic of yogurt-like sensory notes [51]. These metabolic changes contribute to improved aroma complexity and product acceptability depending on strain selection and fermentation conditions.

For example, Mertdinc et al. [48] investigated PBB derived from two geographically distinct pistachio varieties cultivated in Turkey. The authors reported that catechin and gallic acid were the predominant phenolic compounds. In addition, in vitro analyses indicated bioaccessibility values exceeding 20% for phenolic compounds and approximately 200–205% for antioxidant activity, suggesting enhanced release of bioactive constituents following digestion simulation. Sensory evaluation yielded acceptability scores ranging from 5.6 to 7.0 on a 10-point hedonic scale, indicating moderate consumer acceptance.

Reale et al. [51] developed fermented pistachio beverages using multiple LAB strains, including *Leuconostoc pseudomesenteroides* PD4, *L. plantarum* PT1 and PV-2, *Companilactobacillus kimchi* PU2, *Companilactobacillus alimentarius* PG3, and *Lactiplantibacillus paraplantarum* PN4. Fermentation resulted in high microbial viability, with probiotic cell populations exceeding 8 log CFU/mL. Moreover, the fermented products maintained probiotic stability under refrigerated storage (4 °C) for up to 30 days, remaining above the commonly accepted functional threshold for probiotic foods.

Gonçalves et al. [52] evaluated a water-soluble pistachio beverage fermented with water-kefir grains, supplemented with a sucrose/cocoa honey blend and pumpkin seed protein. The formulation led to a substantial increase in kefir grain biomass (approximately 309%), accompanied by sustained viability of LAB and yeasts. Fermentation also enhanced antioxidant activity, highlighting the role of symbiotic microbial consortia in improving functional properties of pistachio-based matrices.

Di Renzo et al. [50] further demonstrated that selected starter cultures, including *L. pseudomesenteroides* and *Companilactobacillus paralimentarius*, exhibit rapid growth kinetics in pistachio substrates, reaching microbial concentrations between 8 and 10 log CFU/mL within 24 h of fermentation. These findings confirm the suitability of pistachio milk as a growth-supporting matrix for LAB proliferation.

Beyond microbial and sensory modifications, fermentation induces deeper biochemical transformations in pistachio milk. Although a reduction in radical scavenging activity has been reported in some cases (up to approximately 40%), this effect is counterbalanced by the formation of bioactive metabolites during proteolysis and microbial metabolism [53]. In particular, LAB contribute to the release of bioactive peptides with reported in vitro antihypertensive and hypoglycemic potential, indicating that fermentation acts as a bioconversion process enhancing the functional value of the matrix.

Furthermore, the nutritional and bioactive profile of pistachio-based beverages is strongly influenced by pre-fermentation processing. Mechanical treatments such as colloidal milling reduce particle size, disrupt plant tissues, and improve matrix homogenization, facilitating the release and dispersion of nutrients and bioactive compounds while enhancing beverage stability [3,8,54]. The resulting fine emulsions may improve the accessibility of lipophilic compounds, including carotenoids and vitamin E, during digestion [9]. In addition, pistachio-based fermented beverages have shown promising nutritional characteristics, including favorable amino acid profiles, supporting their potential as functional PBBs [49].

Overall, these findings indicate that pistachio milk represents a promising substrate for the development of fermented PBBs, offering potential improvements in microbial viability, bioactive compound modulation, and sensory complexity, although outcomes remain highly dependent on strain selection and processing parameters.

Table 6 summarizes these findings in terms of strain viability, antioxidant activity and phenolic content.

### 3.5. Walnut Milk (Juglans regia)

Walnut milk (*Juglans regia* L.) is distinguished among PBMAs by its high content of omega-3 fatty acids, particularly α-linolenic acid, as well as its richness in PUFAs and phenolic compounds such as ellagic acid and ellagitannins [11]. These compositional features contribute to its strong nutritional and functional potential. However, its application in commercial formulations is often limited by the presence of undesirable grassy and oxidized flavor notes, which are associated with volatile aldehydes. Targeted fermentation processes have been shown to modulate these volatile compounds, thereby improving sensory quality while preserving the functional properties of the matrix [11,55].

Under optimized fermentation conditions, walnut-based substrates support the growth of probiotic cultures. Previous studies have reported VCCs around 6 log CFU/mL for strains including *L. plantarum*, *B. breve*, *L. rhamnosus*, *S. thermophilus*, and *L. delbrueckii* subsp. *bulgaricus* in fermented walnut matrices [56]. These findings indicate that walnut milk provides a suitable environment for LAB survival and activity.

Zhong et al. [57] evaluated the fermentation of walnut-based substrates using *L. paracasei* SMN-LBK to enhance antioxidant peptide production. Under optimized conditions (5% inoculum, 37 °C, 12 h fermentation), the resulting system exhibited strong antioxidant activities, including DPPH radical scavenging (77%), ABTS scavenging (93%), and metal ion chelating capacity (79%). These results highlight the role of fermentation in generating bioactive peptides with functional potential in food systems.

Li et al. [58] investigated walnut milk fermentation using *L. plantarum* strains applied before and after proteolysis. VCCs ranged from 9.46 to 9.97 log CFU/mL, indicating strong microbial growth. They demonstrated through untargeted metabolomics that fermentation substantially remodeled the metabolite profile of walnut milk, increasing the abundance of amino acids, peptides, organic acids, and other bioactive metabolites associated with improved antioxidant properties. Consistent with these findings, Liu et al. [55] reported enhanced functional characteristics and flavor quality following *L. plantarum* fermentation, including increased antioxidant activity and reduced off-flavor aldehydes.

Ma et al. [59] developed a dual-protein walnut-based yogurt using *L. plantarum* JLAU103. The product demonstrated high microbial stability during 21 days of storage at 4 °C, with VCCs consistently exceeding 7 log CFU/mL. In addition to microbial robustness, the fermented product exhibited improved sensory acceptance compared to dairy controls, attributed to a complex volatile profile containing over 200 compounds, including aldehydes and furans with desirable nutty aromas. Fermentation also enhanced antioxidant activity through the generation of low-molecular-weight (<3 kDa) bioactive peptides with strong radical scavenging capacity.

Cui et al. [60] studied kefir grain fermentation of walnut milk and identified optimal conditions at 30 °C for 12 h with 8% sucrose supplementation. Under these conditions, the beverage achieved favorable sensory properties, including improved aroma balance and reduced off-flavor perception. Microbiological analysis showed high viability of LAB (8.04 log CFU/mL), lactococci (7.91 log CFU/mL), and yeasts (6 log CFU/mL), indicating a stable and active fermentation system. In a related study, Cui et al. [61] developed a fermented beverage based on a chestnut–walnut protein blend using a co-culture of *L. rhamnosus* and *L. casei*. The fermentation process resulted in a high probiotic population reaching 9.15 log CFU/mL. Additionally, TPC increased from 11.10 to 20.21 mg/L, accompanied by a 32% increase in DPPH radical scavenging activity. The fermentation also generated 36 new volatile compounds, contributing to improved aroma complexity and reduced off-flavor intensity.

Finally, Liu et al. [55] demonstrated that short-term fermentation of walnut milk with *L. plantarum* LP56 significantly modifies its biochemical and sensory profile. Fermentation reduced aldehyde-associated off-flavors while increasing desirable volatile compounds such as alcohols, esters, and ketones. In addition, levels of essential amino acids and unsaturated fatty acids increased, reaching 2775 mg/L, thereby improving both nutritional and sensory quality. These findings suggest that fermentation acts as a metabolic modulation strategy to enhance the overall quality of walnut-based beverages.

Overall, the literature indicates that walnut milk is a highly suitable substrate for fermentation-driven development of functional PBBs, with improvements in microbial viability, antioxidant activity, volatile profile modulation, and sensory acceptability, depending on strain selection and processing conditions.

Table 7 presents these findings in terms of strain viability and antioxidant activity.

## 4. Discussion

The industrial production of PBBs involves the transformation of complex plant matrices into physically stable colloidal systems, requiring a balance between structural stability, nutritional retention, and sensory quality [9]. Conventional mechanical disintegration remains widely used; however, emerging processing strategies such as high-pressure homogenization and enzymatic treatments (e.g., with cellulases and proteases) have significantly improved extraction efficiency and matrix functionality [3,6,7,8]. Despite these advances, processing inevitably alters the nutritional composition. For example, reductions in bioactive compounds such as phenolics and antioxidant components have been reported during mechanical or thermal treatments, highlighting the balance between stability and nutritional integrity [3,8]. Unlike cow’s milk, which has a naturally stable colloidal structure shaped by evolutionary adaptation, raw nut matrices are highly unstable, polydisperse systems prone to rapid protein flocculation and gravitational phase separation [9].

Thermal treatments such as pasteurization and UHT processing are essential for the microbial safety of the products but can negatively affect the nutritional value (e.g., heat-sensitive vitamins and phytochemicals), reinforcing the need for nutrient fortification (e.g., Ca, vitamin D, and B-complex vitamins) [3,7,8,10,12,13]. In this context, non-thermal technologies such as thermosonication and high-pressure processing have emerged as promising alternatives, as they better preserve amino acids, phenolics, and antioxidant compounds [6,7,8,10,12]. Additionally, hydrocolloids (e.g., xanthan gum) and emulsifiers (e.g., lecithin) are commonly employed to improve viscosity, emulsification, and physical stability by preventing phase separation and sedimentation [3,7,8,10,12]. Although both types of additives contribute to the physical stability of PPBs, they act through different mechanisms.

Despite their functional potential, NBBs generally exhibit lower intrinsic nutritional density compared to bovine milk. While cow’s milk is widely used as a nutritional benchmark due to its balanced composition and high protein digestibility [3,8,13,16], PBMAs typically contain lower protein levels and may lack certain essential amino acids such as lysine and methionine depending on the raw material [6,11,16]. From a quantitative and qualitative standpoint, cow’s milk is characterized by high protein density (~3.2–3.4 g/100 mL) and a well-balanced indispensable amino acid profile, which together contribute to consistently high protein quality scores, including Protein Digestibility-Corrected Amino Acid Score (PDCAAS) values near or at 1.0 and Digestible Indispensable Amino Acid Score (DIAAS) values often exceeding 100% for growth-supporting amino acid requirements [62,63]. In contrast, most NBBs are substantially lower in protein concentration and are typically limited by essential amino acids such as lysine, resulting in reduced overall protein quality, with soy-based formulations generally performing better than nut-derived alternatives [62,63].

To improve the nutritional profile of PBMAs, formulation strategies such as the combination or selection of complementary plant protein sources are discussed in the literature as potential approaches to improve amino acid balance and overall protein quality in plant-based systems [63]. Nevertheless, plant-based systems provide distinct advantages, particularly their cholesterol-free lipid profiles and PUFA contents, which contribute to improved cardiovascular relevance [8]. Furthermore, PBMAs naturally contain bioactive compounds including tocopherols, dietary fiber, phenolics, and other antioxidant constituents, positioning them as functional delivery systems beyond simple dairy substitutes [10,11,14,15].

Fermentation further enhances the functional potential of NBBs by introducing probiotic microorganisms and enabling biochemical transformations that improve both nutritional and sensory properties. LAB, particularly species of *L. plantarum*, *L. rhamnosus*, and related taxa, are frequently employed due to their adaptability to plant matrices and probiotic functionality, achieving viabilities in the range 6–9 log CFU/mL [7,11,13,17]. The enhanced functionality through fermentation is also associated with microbial proteolysis that releases bioactive peptides, modifies phenolic availability, and generates organic acids and other metabolites with improved antioxidant activity [55,58]. Untargeted metabolomic analyses have further identified and confirmed these substantial compositional changes during fermentation, ref. [58].

The nutritional optimization by fermentation also depends on the reduction in anti-nutritional factors (ANFs), which differ among nut species. Almonds and walnuts are primarily limited by phytic acid and tannins, hazelnuts by trypsin inhibitors, and coconuts and pistachios by soluble oxalates. Phytic acid and oxalates chelate divalent Fe, Zn, and Ca, reducing intestinal absorption, while tannins inhibit digestive proteases [64]. LAB fermentation improves nutrient availability by modifying the substrate composition and reducing ANFs through both microbial activity and processing conditions. For example, acidification and microbial metabolism can contribute to the reduction in certain ANFs such as protease inhibitors, or phytase production in certain strains, may contribute to enhanced mineral bioavailability (e.g., Fe, Zn, Ca). These fermentation effects are strain-, substrate-, and processing-dependent [7,8,17]. Finally, although fermentation can improve the nutritional and functional properties of PBBs, it does not fully eliminate the nutritional differences relative to bovine milk, making fortification and formulation strategies relevant for improving nutritional adequacy [62,63,65,66]. However, the structural shifts in substrate components by microbial metabolism that improve antioxidant capacity and bioaccessibility of micronutrients may protect the probiotic cells during subsequent digestion, where extreme gastric conditions (pH 1.5–2.0) and bile salts can substantially reduce their viability [67].

Matrix-specific differences are particularly evident among nut-based systems. Almond milk is among the most extensively studied nut matrices [19,21,25,27,28]. In comparison, fewer studies are available on pistachio and hazelnut-based beverages [41,42,43,44,45,46,47,48,49,50,51], which limits direct comparison of probiotic survival and sensory outcomes across these systems. Studies have demonstrated that almond-based matrices can sustain probiotic viability above 6–8 log CFU/mL during fermentation and refrigerated storage, and show improved sensory acceptance and antioxidant activity after fermentation [19,21,25,27,28]. This high survival of probiotic lactobacilli may be attributed to the favorable nutrient composition of the almond matrix, which supports microbial growth during fermentation [19,21,27,28]. Moreover, the availability of fermentable substrates enhances acid tolerance by supplying energy for membrane-bound F_0_F_1_-ATPase-mediated proton extrusion, thereby helping maintain intracellular pH under acidic conditions [68].

**Figure 1:**
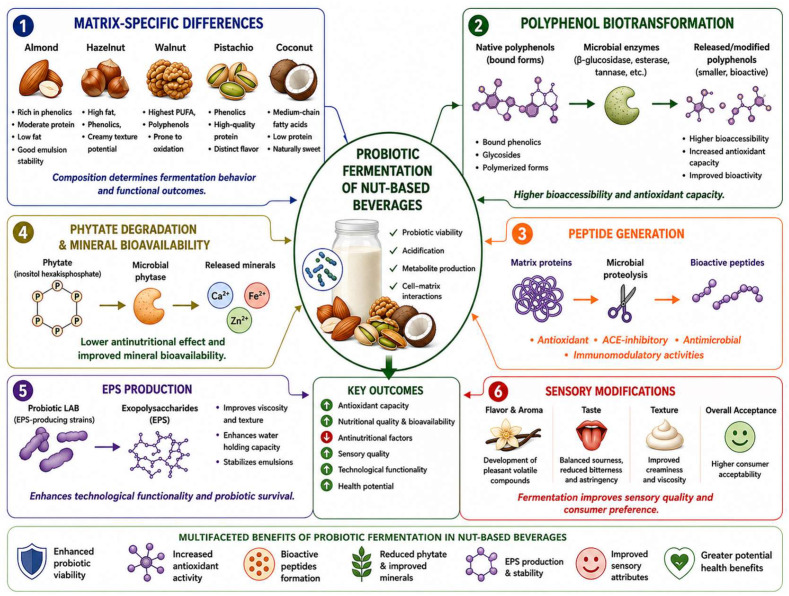
Schematic overview of the matrix-specific characteristics, microbial mechanisms, and technological, nutritional, and sensory outcomes associated with probiotic fermentation of nut-based beverages.

In addition to microbial stability, fermentation is associated with increased TPC and enhanced antioxidant activity, often attributed to microbial enzymatic release of bound phenolics [19,20]. Synbiotic formulations, such as those incorporating inulin, have also been shown to further enhance microbial growth and acidification kinetics without compromising product stability [21]. At the molecular level, these structured prebiotic oligosaccharides form a natural, protective hydro-membrane sheath around the probiotic cell wall, significantly increasing resistance to environmental and gastrointestinal stresses [67]. Moreover, adjunct ingredients such as fruit juices or kefir cultures can improve both sensory acceptability and bioactive potential [25,27]. Technological interventions, including spray-drying microencapsulation, have also demonstrated effectiveness in maintaining probiotic viability over extended storage periods, supporting industrial scalability [24]. Overall, fermented almond beverages demonstrate competitive sensory profiles compared to other PBB systems, though outcomes remain dependent on formulation and microbial strain selection [27,28] (Figure 1).

Pistachio milk is distinguished by its rich content of carotenoids, chlorophylls, phenolic compounds, and high-quality proteins, supporting its application as a functional fermentation substrate [11,48,49]. Fermentation with LAB, including *Leuconostoc* and *Lactiplantibacillus* species, can achieve high microbial densities, exceeding 8 log CFU/mL, with stability during refrigerated storage [50,51]. Although fermentation may initially reduce radical scavenging activity in some cases, which typically happens due to the transient oxidation of free phenolics during aerobic agitation, it simultaneously promotes the formation of bioactive peptides and secondary metabolites with potential antihypertensive and hypoglycemic effects [53]. Although fermentation may initially reduce radical scavenging activity in some cases, antioxidant responses are dynamic and depend on fermentation conditions and microbial biotransformation of phenolic compounds and other bioactive constituents. For example, agitated fermentation may lead to the transient oxidation of free phenolics, while on the other hand, it can promote the release of bound phytochemicals through cell wall degradation, as well as the generation of antioxidant peptides and other bioactive metabolites, resulting in enhanced functional properties, including potential antihypertensive and hypoglycaemic effects [53,69]. Additionally, fermentation alters volatile compound profiles by reduction in grassy notes and the emergence of more dairy-like characteristics, contributing to improved sensory quality, given that pronounced raw plant-derived aromas are considered a major barrier to consumer acceptance of PBMAs [1,51]. Advanced processing methods such as colloidal milling further improve nutrient release and phenolic bioaccessibility, strengthening the functional value of pistachio-based beverages [49,51]. Such high-shear homogenization generates fine oil-in-water emulsions by reducing oil droplet size, thereby improving emulsion stability and reducing the tendency for cream separation during storage. These structural modifications can also enhance mouthfeel and provide an effective delivery system for fat-soluble bioactive compounds [9].

Overall, the available evidence indicates that almond and pistachio matrices provide favorable environments for probiotic growth while supporting desirable sensory and functional improvements following fermentation [19,20,21,25,27,28,48,49,50,51,52,53] (Figure 1). However, these apparent differences should be interpreted cautiously because relatively few studies have compared multiple nut matrices under identical experimental conditions. As a result, the superior performance reported for certain matrices may partly reflect differences in starter cultures, formulation strategies, and processing parameters rather than intrinsic properties of the nut substrate alone.

In contrast, the physical stability of coconut-based beverages can benefit from additional structural stabilization during processing and storage. Fermentation with EPS-producing LAB has been shown to increase viscosity and improve textural properties, while contributing to overall stability [29,33,38]. These microbial glucan- and fructan-type EPS enhance the viscosity of the continuous phase and promote the formation of a more cohesive structural network, thereby reducing phase separation and improving emulsion stability [33,38]. Across multiple studies, probiotic viability generally remains above 6 log CFU/mL during fermentation and refrigerated storage [29,32,33,35,37]. Fermentation with LAB such as *L. plantarum*, *L. acidophilus*, and *S. salivarius* has been associated with improved antioxidant activity and increased TPC, alongside enhanced antimicrobial properties [30,34,35,37]. Sensory properties of fermented coconut beverages can be optimized through careful selection of probiotic strains and formulation adjustments, including sweetness optimization, to balance acidity and improve consumer acceptability [29,37]. Maintaining an appropriate balance between acidity and sweetness is important for preserving favorable sensory attributes throughout storage, as excessive acidification may reduce overall consumer acceptance [29,34]. In addition, the choice of starter culture and fermentation conditions can influence the sensory profile, supporting the development of products with improved consumer appeal [29,32,37]. Collectively, fermented coconut-based beverages represent promising systems for functional food development due to their strong probiotic compatibility and bioactive enhancement potential [35,37,38].

Hazelnut milk is characterized by a high content of monounsaturated fatty acids and vitamin E, making it a nutritionally relevant matrix for fermentation [7,11,39]. Studies indicate that hazelnut-based substrates can effectively support LAB growth, with probiotic counts typically exceeding 6 log CFU/mL during refrigerated storage [41,42,46]. Fermentation using kefir grains or yogurt cultures enhances antioxidant activity, in some cases nearly doubling radical scavenging capacity due to the formation of bioactive metabolites [44,47]. Structural improvements have also been reported, including increased creaminess and gel stability, attributed to microbial polysaccharide production and protein/polysaccharide interactions [45]. However, sensory acceptance may vary depending on processing conditions, with optimized fermentation parameters (e.g., lower incubation temperatures) helping preserve characteristic nutty aromas and improve consumer acceptability [41,47]. For example, during LAB fermentation of hazelnut milk, acidification leads to a pH drop to about 4.9 and significant changes in texture and rheological behavior, including pseudoplastic flow characteristics and increased structural consistency [65]. These transformations highlight the strong influence of fermentation on the stability and sensory profile, and adjusting fermentation kinetics to avoid overacidification may serve as a suitable mitigation strategy to protect the beverage’s commercial value [67].

Lipid oxidation is a key quality challenge in PPBs, contributing to the formation of volatile compounds associated with rancid off-flavors, while fermentation can help improve sensory properties and product acceptability through the metabolic activity of LAB [1]. Walnut milk is characterized by high levels of α-linolenic acid and phenolic compounds, although its sensory acceptance can be limited by the development of lipid-derived volatiles associated with oxidation effects on unsaturated fatty acids, which may contribute to off-flavor perception during storage and processing [9,11,55]. Fermentation with LAB such as *L. plantarum* effectively modulates volatile profiles by reducing aldehydes and increasing desirable esters, alcohols, and ketones, thereby improving aroma quality and overall acceptability [55]. Moreover, walnut-based matrices support strong probiotic growth, typically achieving VCCs in the range of 7–9 log CFU/mL depending on strain and fermentation conditions [58,59,60,61]. Fermentation also enhances the functional properties through proteolytic activity, which releases bioactive peptides and increases TPC, resulting in improved antioxidant and metal-chelating capacities [57,58,61]. Pre-fermentation enzymatic and fermentation-related protein modifications can influence peptide formation and improve antioxidant activity in food systems, contributing to enhanced oxidative stability during processing [68]. In this respect, kefir and mixed-culture fermentations in NBB systems have been shown to influence physicochemical properties and flavor profiles in a strain-dependent manner, contributing to variations in sensory characteristics [60,61].

Overall, fermentation is a key technological strategy for improving the nutritional, functional, and sensory properties of NBBs. However, although the overall effects are generally positive, direct comparison among studies remains challenging due to substantial methodological heterogeneity, limiting cross-study comparability and industrial standardization. Specifically, the available studies differ considerably in substrate composition (nut concentration and other constituents), thermal pretreatment, probiotic strains used, inoculum levels, fermentation time, storage conditions, and analytical methodologies used to evaluate antioxidant activity, phenolic content, and sensory quality. Consequently, it is often difficult to distinguish matrix-specific effects from those arising from differences in fermentation protocols, limiting quantitative comparison across studies and highlighting the need for standardized experimental designs.

Regarding increases in TPC and antioxidant activity that are frequently reported following fermentation, the level and nature of these changes are not entirely consistent across studies. Such variability also reflects differences in microbial strain metabolism, fermentation duration, oxygen exposure, substrate composition, and the analytical assays employed. In several studies, transient reductions in antioxidant activity during early fermentation were followed by subsequent increases associated with microbial metabolism and the formation of bioactive metabolites, suggesting that antioxidant responses are dynamic rather than uniform across fermentation systems [53,55,58,69].

Table 8 provides an integrated comparison of NBBs across nut matrices, based on the available literature, including probiotic viability, antioxidant enhancement, sensory acceptance, technological challenges, and industrial potential.

In parallel, the industrial development of NBBs is influenced by regulatory requirements, food safety considerations, and compositional limitations. Plant-based matrices naturally contain ANFs such as phytates and trypsin inhibitors, which can reduce mineral bioavailability and digestive efficiency [64,66]. Processing such as fermentation and enzymatic treatment can partially reduce ANFs and improve nutritional functionality, although full equivalence with cow’s milk is not typically achieved [18,64]. Regulatory frameworks governing PBMAs vary internationally and generally emphasize clear product labeling to avoid consumer confusion regarding dairy terminology. In the United States, general FDA food labeling and misbranding provisions emphasize accurate product characterization and consumer transparency rather than assigning a distinct regulatory category specific to PBMAs (21 CFR §101.3). Internationally, nomenclature is further guided by the Codex Alimentarius General Standard for the Use of Dairy Terms [70]) to prevent consumer confusion. European Union legislation under Regulation (EU) No 1308/2013 restricts the use of protected dairy terms such as “milk” to mammary secretions, reinforcing origin-based classification, with limited exceptions shaped by historical usage. Therefore, fermentation and other processing technologies must balance functional improvement with compliance with compositional safety and labeling requirements, underscoring the multidimensional constraints governing PBMA development and commercialization.

Beyond technological parameters, PBMA industrialization is also shaped by environmental and economic constraints evaluated through lifecycle concepts. Compared with cow’s milk, PBMAs generally show lower greenhouse gas emissions and land-use impacts, although outcomes vary by raw material and processing type [15,16,66]. However, optimization challenges remain due to resource inputs and manufacturing demands, requiring broader sustainability assessment frameworks [66]. Economically, the high raw material costs and processing requirements remain key barriers to scalability, motivating improved process efficiency and by-product valorization strategies within PBMA production systems [6,13,66].

Finally, it should be taken into account that nuts and NBBs present several intrinsic limitations related to safety, stability, and functionality. Tree nuts are recognized as major food allergens, requiring strict management across processing and fermentation systems, as structural protein modifications do not fully eliminate allergenic risk [13,16]. Their high unsaturated lipid content also makes them susceptible to oxidative deterioration that may negatively affect both quality and stability [15,66,69]. In addition, nuts may be vulnerable to microbial and chemical hazards, including contamination by pathogenic bacteria and mycotoxins under inadequate storage conditions [13,16]. Maintaining probiotic viability in fermented NBBs remains a further challenge, as it is also influenced by matrix composition, oxygen exposure, and storage conditions, making sustained functional cell counts difficult to ensure over shelf life [19,21,24,29,33,41,50,58].

Overall, the available evidence consistently supports the feasibility of producing probiotic fermented NBBs with acceptable microbial viability and improved functional properties. However, much of the current literature is based on laboratory-scale studies using different formulations, microbial strains, processing conditions, and analytical protocols, limiting direct comparison across studies. Consequently, further validation under standardized and commercially relevant processing, storage, and sensory evaluation is needed to facilitate industrial translation of fermented, probiotic NBBs.

Future research should focus on strain-specific metabolic pathways, microbial–matrix interactions (e.g., phenolics biotransformation, EPS biosynthesis, peptide release mechanisms, etc.) and enzymatic transformation mechanisms that govern the nutritional, functional, and sensory properties of fermented NBBs. The integration of metabolomics, proteomics, and transcriptomics with conventional physicochemical analyses may provide a more comprehensive understanding of microbial metabolism and fermentation dynamics, facilitating the rational selection of starter cultures and the optimization of fermentation processes. In addition, the development of tailored mixed-culture fermentations exploiting complementary metabolic activities among LAB and other beneficial microorganisms represents a promising strategy to improve flavor complexity, texture, nutritional quality, and probiotic functionality in fermented PBBs [6,14,17,18,22,66]. Particular attention should also be given to controlled comparative studies evaluating different nut matrices using identical starter cultures and fermentation conditions, thereby allowing matrix-specific effects to be distinguished from strain-dependent responses.

## 5. Conclusions

The development of NBBs as dairy alternatives represents an important advancement in functional beverage science and technology. Although NBBs generally contain lower protein levels than cow’s milk, they provide valuable bioactive compounds, including unsaturated fatty acids, dietary fiber, and antioxidants. Advanced processing technologies such as high-pressure homogenization and non-thermal treatments play a key role in improving structural stability while preserving heat-sensitive nutrients and bioactive compounds. Fermentation with LAB significantly enhances the functional and sensory properties of NBBs by increasing microbial viability (6–9 log CFU/mL), improving phenolic release, antioxidant activity, volatile profile, and mineral bioaccessibility. These effects are strongly dependent on microbial strain, substrate composition, and fermentation conditions.

Among nut matrices, almond and pistachio-based beverages generally exhibit more consistent fermentation performance, supporting high probiotic viability and improved sensory and functional outcomes. In contrast, coconut-based beverages often require additional structural stabilization due to limited intrinsic emulsifying capacity, while walnut-based systems are more prone to oxidative instability and off-flavor development despite their high nutritional value. Hazelnut beverages show intermediate behavior with favorable lipid composition and improved antioxidant properties after fermentation.

Overall, standardization of processing conditions remains essential for improving scalability and comparability across studies. Future research should focus on strain-specific metabolic pathways and microbial–matrix interactions using integrated omics approaches, alongside predictive fermentation models for process optimization.

Finally, although standardized comparative studies are valuable for distinguishing matrix-specific effects under controlled fermentation conditions, future research should not be directed solely toward identifying a single “optimal” nut matrix. Each nut possesses distinct compositional, nutritional, and sensory characteristics that influence its fermentation behavior and potential applications. Given the diversity of consumer preferences, dietary requirements, allergenicity, cultural practices, and regional availability, future research should aim to optimize fermentation strategies for individual nut matrices rather than prioritize one substrate over another. Such an approach would better support the development of a diverse portfolio of fermented NBBs tailored to different consumer needs and market demands.

## Figures and Tables

**Table 1:** Influence of specific pretreatment and extraction strategy on the nutritional, structural and functional properties of NBBs.

Beverage	Pretreatment/Processing	Impact on Nutritional Composition	Techno-Functional Effects	Ref.
Almond	Roasting (95–100 °C)	Alters the sterol profile (increases β-sitosterol-β-D-glucoside; decreases stigmasterol).	Reduces concentrations of benzaldehyde and pyrazines to develop desirable aroma.	[6,7,8,10]
	Thermosonication (45 °C; 40 min)	Does not degrade amino acids; preserves the nutritional composition.	Improve self-life and stability.	
	UHPH (350 MPa; 85 °C)	Eliminates the allergenic potential of almond proteins.		
	PEF	Successfully preserves heat-sensitive antioxidants and vitamins.	Effectively inactivates spoilage microorganisms and quality-degrading enzymes; no thermal damage.	
Coconut	Blanching or steam cooking; aqueous extraction (80 °C; 10 min)	Preserves lipid components such as lauric acid and medium-chain triglycerides.	Achieves endogenous enzyme inactivation and microbial reduction.	[7,10]
	Microfiltration (no-heat sterilization)	Serves as a non-thermal alternative that avoids heat-induced chemical degradation.	Efficiently removes microorganisms and increases shelf life.	
	Homogenization		Ensures structural stability; enhances the milk white color and clarity.	
	0.3% pectin + 30 ppm SO_2_ + homogenization (13,000 rpm; 2 min) + pasteurization (100 °C; 5 min)	nr	Minimizes sedimentation; provides emulsion stability; significantly lowers pH; eliminates yeasts, molds, and bacteria.	
Hazelnut	Roasting (95–100 °C)	nr	Develops aroma.	[6,8,10,12]
	Aqueous extraction	Significantly reduces TPC.	nr	
	Advanced thermosonication (60% amplitude; 25 min or 80% amplitude; 15 min) or homogenization (100 MPa)	Better retains amino acids and bioactive compounds compared to conventional heat treatments.	Eliminates microorganisms (yeasts, molds, total aerobic bacteria); optimizes structure (minimizes syneresis/sedimentation, improves consistency and viscosity).	
Pistachio	Grinding soaked kernels + blending in hot water (80 °C) with traditional filtration	nr	Generates significant waste (fibrous outer skins) during filtration.	[7,11]
	Milling roasted kernels in a colloid mill (3000 rpm; 5–10 min) at pH 8.5 and thermal treatment (70 °C)	Increases protein, fat, dry matter, and total soluble solids. Colloid milling with recirculation ensures richness in essential amino acids and releases bioactive peptides with ACE and DPP-4 inhibition activities.	Generates no processing waste; yields a physically stable beverage with minimal sedimentation; minimizes microbial counts; provides the highest sensory acceptability.	
Walnut	Chemical pellicle removal (using 2% citric acid; 90 °C; 2–3 min or 1% NaOH; 90 °C; 10 min)	Results in loss of antioxidant activity, as the majority of antioxidants are concentrated in the skin.	Reduces bitterness caused by tannins and other phenolics, improving sensory properties.	[7,8]
	Two-stage homogenization (40 MPa) and/or high-temperature thermal treatment (120 °C; 10 min)	nr	Two-stage homogenization breaks down large droplets but they tend to flocculate.	
			High-temperature thermal treatment increases particle size and forms larger oil droplet aggregates.	
			Combining both methods further increases particle size, resulting in more floating layer and less precipitate.	

UHPH: Ultra-high-pressure homogenization. PEFs: Pulsed Electric Fields. TPC: Total phenolic content. nr: not reported. ACE: Angiotensin-Converting Enzyme. DPP-4: Dipeptidyl Peptidase IV.

**Table 3:** Fermented almond milk beverages in terms of probiotic viability, phenolic content, and antioxidant activity.

Probiotic Strains	Strain Viability (log CFU/mL)	TPC (mg GAE/mL)	TFC	Antioxidant Activity	Ref.
*L. rhamnosus*; *L. acidophilus*; *L. plantarum*; *L. casei*	~6	Increased	Increased (highest with *L. rhamnosus*)	Increased	[19]
*L. rhamnosus*; *L. acidophilus*; *L. plantarum*; *L. casei* in almond/cow milk blends	5.9–6.8	Increased	Increased	Increased	[20]
*L. rhamnosus* GR-1 + short- and long-chain inulin	≥7	nr	nr	nr	[21]
*L. plantarum* ATCC 8014	>7	nr	nr	nr	[24]
*L. acidophilus* + orange juice supplementation	7.58–7.95	nr	nr	nr	[25]
Kefir grains (LAB consortium)	8	0.95	nr	Increased (62–66% DPPH inhibition)	[27]
*S. thermophilus*; *L. delbrueckii* subsp. *bulgaricus*; *L. acidophilus* NCFM; *B. lactis* HM019^TM^	>6 (except *B. lactis*)	nr	nr	Increased (0.72–0.75 mmol TE/L)	[28]

LAB: Lactic acid bacteria. CFU: Colony-forming units. TPC: Total phenolic content. TFC: Total flavonoid content. GAE: Gallic acid equivalent. TE: Trolox equivalents. DPPH: 2,2-diphenyl-1-picrylhydrazyl radical. nr: not reported.

**Table 4:** Fermented coconut milks in terms of strain viability, antioxidant activity and phenolic content.

Probiotic Strains	Strain Viability (log CFU/mL)	TPC	TFC	Antioxidant Activity	Ref.
*B. animalis* B-41406; *B. bifidum* B-41410; *B. breve* B-41408; *B. longum* subsp. *infantis* B-41661	>6 (up to 42 days, 4 °C)	nr	nr	nr	[29]
*S. salivarius *ATCC 13419 , *K12*; *L. casei *ATCC 393; *L. rhamnosus *ATCC 53103; *L. acidophilus *ATCC 314; *L. fermentum *ATCC 14931; *L. fructivorans*	*S. salivarius*, highest among tested strains	nr	nr	Up to 67% inhibition against *S. pyogenes* depending on strain	[30]
*L. paracasei *MSMC 36-9	12–13 (21 days, 4 °C)	nr	nr	Higher antioxidant activity than commercial yogurt starter culture	[32]
*L. plantarum* SVP2	~8 (7 days, 4 °C)	nr	nr	nr	[33]
*L. casei*; *L. plantarum*; *L. rhamnosus*; *Lactococcus lactis* IO-1	Up to 8.4 (during storage, 4 °C)	Increased during storage	nr	Increased during storage	[34]
*L. acidophilus 10307*	6.32 (28 days, 4 °C)	7.8–14 mg GAE/mL	26.5–87.7 mg QE/mL	Strong radical scavenging (DPPH, H_2_O_2_)	[35]
*L. plantarum *CMGC2, CMJC7	>8 (48 h fermentation)	nr	nr	nr	[36]
*L. plantarum *DW12	8.4 (48 h fermentation)	134 μg GAE/mL	nr	ABTS 75%; DPPH 55%	[37]

CFUs: Colony-forming units. TPC: Total phenolic content. TFC: Total flavonoid content. GAE: Gallic acid equivalent. QE: Quercetin equivalent. ABTS: 2′-azino-bis(3-ethylbenzothiazoline-6-sulfonic acid) radical. DPPH: 2,2-diphenyl-1-picrylhydrazyl radical. nr: not reported.

**Table 5:** Fermented hazelnut milks in terms of strain viability, antioxidant activity and phenolic content.

Probiotic Strains	Strain Viability (log CFU/mL)	TPC	Antioxidant Activity	Ref.
Viili microbiota	Up to 7.91	nr	nr	[41]
*L. rhamnosus* GG	7.9–8.3 (28 days, 4 °C)	nr	nr	[42]
Kefir grains	9.5% decrease in LAB during storage	nr	nr	[43]
Yogurt culture (LAB)	>6 (per g or mL depending on matrix)	nr	nr	[45]
*L. acidophilus* (frozen dessert system)	3.22–8.99	42.3–44.7 (mg GAE/g)	43.8–72.2 mM TE	[46]
Kefir grains	nr	91.8 mg GAE/100 mL	Increased DPPH activity (50.5% → 81.7%)	[47]

LAB: Lactic acid bacteria. CFUs: Colony-forming units. TPC: Total phenolic content. GAE: Gallic acid equivalent. TE: Trolox Equivalent. DPPH: 2,2-diphenyl-1-picrylhydrazyl radical. nr: not reported.

**Table 6:** Fermented pistachio milks in terms of strain viability, antioxidant activity and phenolic content.

Probiotic Strains	Strain Viability (log CFU/mL)	TPC	Antioxidant Activity	Ref.
*L. pseudomesenteroides* PD4; *L. plantarum* PT1/PV-2; *C. kimchi* PU2; *C. alimentarius* PG3; *L. paraplantarum* PN4	8	nr	nr	[51]
Water kefir grains (LAB and yeasts consortium)	nr	Increased	Increased	[52]
*L. pseudomesenteroides*; *C. paralimentarius*	8–10	nr	nr	[50]
Proteolytic LAB strains	nr	nr	40% reduction in radical scavenging	[53]
Commercial yogurt starter culture	nr	nr	nr	[49]

LAB: Lactic acid bacteria. CFUs: Colony-forming units. TPC: Total phenolic content. nr: not reported.

**Table 7:** Fermented walnut milks in terms of strain viability, antioxidant activity and phenolic content.

Probiotic Strains	Strain Viability (log CFU/mL)	TPC (mg GAE/L)	Antioxidant Activity	Ref.
*L. paracasei* SMN-LBK	nr	nr	77% DPPH; 93% ABTS; 79% metal chelation	[57]
*L. plantarum* (before/after proteolysis)	9.46–9.97	nr	Increased after proteolysis	[58]
*L. plantarum* JLAU103	≥7(storage stability)	nr	Enhanced	[59]
Kefir grains	7.91 lactococci; 8.04 lactobacilli; 6 yeasts	nr	nr	[60]
*L. rhamnosus* and *L. casei* co-culture	9.15	11.10 to 20.21 increase	32% increase in DPPH scavenging	[61]

CFUs: Colony-forming units. TPC: Total phenolic content. GAE: Gallic acid equivalent. DPPH: 2,2-diphenyl-1-picrylhydrazyl radical. nr: not reported.

**Table 8:** Comparative synthesis of probiotic fermented nut-based beverages (NBBs): functional performance, technological challenges, sensory outcomes, and industrial feasibility.

Nut Matrix	Probiotic Viability	Functional/Antioxidant Enhancement	Sensory Acceptance	Technological Constraints	Industrial Feasibility
Almond	6–8 log CFU/mL during fermentation and storage [19,21,24,27,28]	Consistent increase in TPC and AA via release of bound phenolics and microbial metabolism [19,20,27,28]	Generally high acceptability, improved flavor profile and reduced raw plant notes [25,27,28]	Moderate emulsion instability; requires homogenization and stabilizers for optimal texture [9,39]	High; well-studied matrix; strong probiotic support; scalable processing
Pistachio	>8 log CFU/mL depending on strain and fermentation conditions [48,49,50,51,52]	Enhanced AA and bioactive peptide formation; responses are strain- and process-dependent [50,53]	Improved sensory quality; reduction in grassy notes; formation of dairy-like volatiles [51,53]	Susceptible to oxidative changes under agitation; emulsion stability depends on processing conditions [49,51]	High; moderate functional matrix requiring process optimization
Walnut	7–9 log CFU/mL [55,58,59,60,61]	Increased AA via peptides and phenolics; strong bioactivity but variable oxidative stability [57,58,61]	Limited acceptability without optimization due to lipid-derived off-flavors [55]	High lipid oxidation susceptibility and volatile rancidity during storage [55,58]	Moderate; nutritionally rich but constrained by oxidative stability
Hazelnut	≥6 log CFU/mL during storage [41,42,47]	Moderate AA and TPC enhancement [44,47]	Moderate to good acceptance; improved creaminess and texture [41,45]	Rheological changes and risk of overacidification affecting stability [65]	Moderate; good lipid profile; requires fermentation control
Coconut	≥6 log CFU/mL; stability with EPS-producing LAB strains [29,33,37]	Increased AA and phenolic release; EPS contributes to functional properties [33,35]	Variable acceptance; strongly dependent on sweetness–acidity balance [29,34]	Low intrinsic emulsifying capacity; phase separation unless structurally stabilized [33,38]	Moderate; requires hydrocolloid/EPS-assisted stabilization
General NBBs	6–9 log CFU/mL; strain-dependent [19,24,29,41,50,58]	Fermentation enhances phenolics, peptides, and AA; effects are highly variable [53,55,58]	Sensory outcomes highly variable; improvement linked to volatile modulation and formulation strategy [22,27]	Key limitations: emulsion instability, oxidation, ANFs, shelf-life variability, and lack of standardization [6,64,66]	Promising; needs standardization; limited by variability and regulatory, safety and sustainability constraints

AA: antioxidant activity. TPC: Total phenolic content. EPS: Exopolysaccharide. ANFs: Antinutritional factors. Values and classifications represent a qualitative synthesis of trends reported across heterogeneous studies. Reported probiotic viability, functional enhancement, and sensory outcomes vary depending on microbial strains, fermentation conditions, substrate composition, and analytical methodologies. Therefore, the comparative categories should not be interpreted as absolute quantitative rankings.

## Data Availability

No new data were created or analyzed in this study. Data sharing is not applicable to this article.

## Abbreviations

The following abbreviations are used in this manuscript:

ABTS	2′-Azino-bis(3-ethylbenzothiazoline-6-sulfonic acid)
CAGR	Compound annual growth rate
CFUs	Colony-forming units
DPPH	2,2-Diphenyl-1-picrylhydrazyl
EPSs	Exopolysaccharides
GAE	Gallic acid equivalent
LAB	Lactic acid bacteria
NBB	Nut-based beverage
PBB	Plant-based beverage
PBMAs	Plant-based milk alternatives
PEFs	Pulsed electric fields
PUFAs	Polyunsaturated fatty acids
QE	Quercetin equivalent
TE	Trolox equivalent
TFC	Total flavonoid content
TPC	Total phenolic content
UHPH	Ultra-high pressure homogenization
UHT	Ultra-high temperature
VCCs	Viable cell counts
